# The Role of Microbiota in *Drosophila melanogaster* Aging

**DOI:** 10.3389/fragi.2022.909509

**Published:** 2022-05-19

**Authors:** Aranzazu Arias-Rojas, Igor Iatsenko

**Affiliations:** Max Planck Institute for Infection Biology, Berlin, Germany

**Keywords:** drosophila, aging, microbiota, lifespan, dysbiosis, dysplasia, intestinal immunity

## Abstract

Intestinal microbial communities participate in essential aspects of host biology, including nutrient acquisition, development, immunity, and metabolism. During host aging, dramatic shifts occur in the composition, abundance, and function of the gut microbiota. Although such changes in the microbiota are conserved across species, most studies remain descriptive and at most suggest a correlation between age-related pathology and particular microbes. Therefore, the causal role of the microbiota in host aging has remained a challenging question, in part due to the complexity of the mammalian intestinal microbiota, most of which is not cultivable or genetically amenable. Here, we summarize recent studies in the fruit fly *Drosophila melanogaster* that have substantially progressed our understanding at the mechanistic level of how gut microbes can modulate host aging.

## Introduction

Biological aging is a complex, multi-factorial phenomenon regulated by a combination of genetic and environmental factors. Genetic studies in laboratory model organisms identified several conserved host pathways, including the insulin/insulin-like growth factor signaling (IIS) and mechanistic Target of Rapamycin (mTOR), which negatively regulate the lifespan of multiple species (Kapahi et al., 2010; Lapierre and Hansen, 2012; Fernandes and Demetriades, 2021). Environmental perturbations, such as changes in temperature, dietary restriction, and stress, can similarly significantly affect life expectancy, and aging in experimental organisms (Valenzano et al., 2006; Fontana et al., 2010; Tatar et al., 2014; Regan et al., 2016; Dutta et al., 2022; Vakkayil and Hoppe, 2022).

Residing at the interface between the host organisms and the environment, commensal microbial communities participate in multiple essential processes, including development, synthesis of essential vitamins, metabolism, immune system modulation, and defense against pathogens (Nicholson et al., 2012; Sommer and Bäckhed, 2013; Geva-Zatorsky et al., 2017; Pickard et al., 2017; Sannino et al., 2018; Ducarmon et al., 2019; Consuegra et al., 2020).

Not surprisingly, there is accumulating evidence that the gut microbiota also plays a significant role in longevity across species (O’Toole and Jeffery, 2015; Clark and Walker, 2018; Kim and Jazwinski, 2018; Valenzano and Seidel, 2018; Bana and Cabreiro, 2019). For example, studies using different human age cohorts identified differences in microbiota composition across various age groups (Biagi et al., 2016). Overall, several studies found that microbial diversity in the gut declines with age (Yatsunenko et al., 2012; Biagi et al., 2016; Leite et al., 2021). However, health-associated genera, such as *Christensenella*, *Akkermansia*, and *Bifidobacterium*, were consistently found in exceptionally long-lived individuals, like supercentenarians, suggesting their potential life span–promoting effects (Biagi et al., 2016).

Although the composition of microbiota varies among taxa, similar to what has been observed in humans, extensive remodeling of the gut microbial communities during aging has also been identified in several model organisms, such as the nematode *Caenorhabditis elegans* (Cabreiro and Gems, 2013; Han B. et al., 2017), the fly *Drosophila melanogaster* (Clark et al., 2015; Li et al., 2016), the fish *Nothobranchius furzeri* (Smith et al., 2017) and the mouse *Mus musculus* (Langille et al., 2014). Whether such age-related changes in the microbiota are causative or a consequence of the aging of the organism remains a challenging question to be answered.

This review summarizes the insights into the intricate relationship between the gut microbiome and host aging obtained using the *Drosophila* model.

Several features of *Drosophila* microbiota laid the foundations for the successful use of the fruit fly model in microbiome research. The simple taxonomic composition combined with the cultivability and genetic tractability of the members of the fly microbiota enables functional studies and deciphering of the molecular mechanisms of commensal influence on the host physiology (Douglas, 2018; Douglas, 2019; Grenier and Leulier, 2020; Lesperance and Broderick, 2020). A wealth of genetic, genomic, and molecular resources available in *Drosophila* helps in the study of host mechanisms of microbiota control and host factors targeted by the commensals (Hales et al., 2015; Ludington and Ja, 2020). The simplicity of generating and maintaining germ-free, also known as axenic, animals is another particular advantage of the *Drosophila* model. Furthermore, gnotobiotic animals colonized with a standardized microbiota can be easily obtained (Douglas, 2018; Ludington and Ja, 2020).

Not surprisingly, due to its exceptional amenability to the experimental manipulations of the microbiome, *Drosophila* has been extensively used to study the impact of microbiota on various physiological processes, including aging (Kuraishi et al., 2013; Trinder et al., 2017; Gould et al., 2018; Ludington and Ja, 2020; Kong et al., 2021; Hrdina and Iatsenko, 2022).

### Composition and Maintenance of *Drosophila* Microbiota

*Drosophila melanogaster* harbors relatively simple microbial communities (2–30 species) in the laboratory and in the field, represented by only two phyla, *Proteobacteria* and *Firmicutes,* and is dominated by two prominent families, *Acetobacteraceae* and *Lactobacillaceae* and two minor families, *Enterococceae* and *Enterobacteriaceae* (Staubach et al., 2013; Wong et al., 2013; Adair et al., 2018). The most consistently associated species across different studies are *Lactiplantibacillus plantarum*, *Levilactobacillus brevis*, *Acetobacter pomorum*, *A*. *pasteurianus*, and *Enterococcus faecalis* (Broderick et al., 2014; Erkosar and Leulier, 2014; Grenier and Leulier, 2020; Lesperance and Broderick, 2020; Ludington and Ja, 2020). Such a community of lactic acid and acetic acid bacteria reflects the fermentative substrates on which flies feed (Chandler et al., 2011; Broderick and Lemaitre, 2012). Diet (substrate) plays an essential role in shaping the *Drosophila* microbiota as the establishment and maintenance of *Drosophila* intestinal commensals relies on the constant intake of microbes from the diet (Erkosar et al., 2014). The vast majority of the intestinal microbes of the fruit flies cannot stably persist in the gut and must be constantly re-ingested with the food (Blum et al., 2013; Broderick et al., 2014; Storelli et al., 2018). The gut of newly emerged flies is colonized with a low number of microbes. However, these flies acquire their microbiota within the first day of their adult life by ingesting bacteria from the food contaminated by the feces of their parents (Blum et al., 2013; Broderick et al., 2014). Additionally, females transmit their microbiota to the offspring by seeding the eggshells of their progenies. Hatching larvae get colonized by eating the contaminated eggshell and ingesting the microbe-rich food on which the bacteria thrive (Erkosar et al., 2014; Storelli et al., 2018). Such associations between *D. melanogaster*, microbiota, and nutrition likely contribute to the high variability in composition and density of microbiota observed between individual flies reared in the same culture vial. The bacterial load can vary by one log between individual co-housed flies (Broderick et al., 2014). Also, flies that are frequently transferred to sterile food, which prevents the re-ingestion of microbes from the substrate, can lose their microbiota, and become germ-free (Blum et al., 2013; Pais et al., 2018). The transitory nature of *Drosophila* microbiota was established using bacterial isolates from *Drosophila* laboratory stocks. Interestingly, some of the bacterial isolates from wild-caught *D. melanogaster* can stably persist and proliferate in the guts of fruit flies. Such stable association facilitated continuous bacterial spreading to new environment and colonization of the next generation of flies with beneficial bacterium which accelerated the growth of *Drosophila* larvae and enhanced the fertility of adult flies, thus conferring fitness advantages for both partners in an ecological context (Pais et al., 2018). Under laboratory conditions, where both microbes and flies can live perfectly fine independently, the evolutionary pressure to maintain a stable association has likely been lost. In line with this, it was found that the diet, rather than the host, is the major force driving the evolution of symbiotic properties of the prominent fly commensal *L. plantarum* (Martino et al., 2018). Such diet-driven evolution of improved symbiotic properties is an example of by-product mutualism, where the host benefits from the by-products of its bacterial symbiont.

### Influence of Gut Microbiota on *Drosophila* Lifespan

The microbiota of the fruit fly changes in abundance and composition throughout aging (Broderick et al., 2014; Guo et al., 2014; Clark et al., 2015; Li et al., 2016; Salazar et al., 2018). Old flies often harbor a higher bacterial load in the intestine than their young counterparts (Blum et al., 2013; Broderick et al., 2014; Marra et al., 2021a). Considering the reported microbiota perturbations, expectedly, the role of microbiota in *Drosophila* aging has been an active area of research. The most direct approach to examining the influence of the gut microbiota on host longevity is to investigate the impact of microbiota removal using axenic flies. Multiple studies that used such an approach reported conflicting results. The pioneering work of Brummel et al. reported that the lifespan of flies reared axenically was shorter than that of conventionally reared flies (Brummel et al., 2004). This effect could be rescued by exposing the flies to microbes within 2–3 days from eclosion. A follow-up study from Ren showed no impact of microbiota elimination on the lifespan (Ren et al., 2007).

Differences in nutrient conditions between laboratories may contribute to the inconsistent results on the impact of microbiota on fly lifespan. Indeed, the bacterial fly commensal *L. plantarum* (Téfit and Leulier, 2017) and yeast *Issatchenkia orientalis* (Yamada et al., 2015; Keebaugh et al., 2018) can promote fly longevity when flies are reared on an undernutrition diet. Remarkably, the same microbes (*I. orientalis*) that extended the lifespan of the fly under poor nutritional conditions shortened the lifespan when flies were reared on nutrient-rich diets (Keebaugh et al., 2019). This observation raises awareness that the nutritional environment and possibly other environmental factors are important determinants of the effect of commensals on the host physiology. Although Keebaugh et al. have not investigated the underlying mechanisms of the detrimental impact of fly commensals on the lifespan under nutrient-rich conditions, their observation provides experimental support for the “overfeeding hypothesis.” Namely, Lachnit et al. (2019) proposed that overfeeding changes functionality of the intestinal microbiota and increases its activity, resulting in the increase of microbial byproducts that promote disease development. Such a scenario could explain the detrimental effect of *I. orientalis* on *Drosophila* lifespan under a nutrient-rich diet. In addition to the nutritional value, dietary pH has also been shown to have an effect on life expectancy. In fact, flies live longer on an acidic diet, similar to what has been observed in axenic animals, suggesting that this effect may be independent of the microbiota (Deshpande et al., 2015). However, the authors observed a shift in the microbiota composition on alkaline pH diets, and this shift might have exacerbated the deleterious effect of alkaline foods on the lifespan (Deshpande et al., 2015). In addition, chemical stressors present in the food could have an impact on the lifespan of fruit flies via affecting the composition of microbiota. For example, transient exposure of *Drosophila* larvae to low concentrations of oxidants extended adult lifespan by selectively eliminating *Acetobacter* species which were causally linked to lifespan shortening (Obata et al., 2018).

Despite conflicting results of early studies, there is lots of evidence showing that the absence of microbes extends the lifespan of *Drosophila*. Numerous studies that utilized different fly genotypes, various diets (cornmeal, chemically-defined), and different ways of generating axenic flies (bleaching, antibiotic treatment) consistently reported an increase in the lifespan of axenic flies (Petkau et al., 2014; Clark et al., 2015; Galenza et al., 2016; Téfit and Leulier, 2017; Iatsenko et al., 2018; Obata et al., 2018; Lee et al., 2019; Shukla et al., 2021). Therefore, the presence of microbiota can be detrimental to the lifespan of fruit flies. Although the microbiota of *Drosophila* undergoes age-related changes in composition and some members of the microbiota appear to be particularly harmful to the host during aging, the extensive work of Lee e al. showed that the abundance of microbes is a stronger determinant of host lifespan than changes in the composition (Lee et al., 2019; Lee et al., 2022).

Several studies attempted to understand the impact of microbes on fruit fly aging and compared the differences between axenic and conventional flies. Shukla et al. took a transcriptomics approach and discovered that around 70% of age-associated changes in gene expression do not occur in germ-free flies (Shukla et al., 2021). Among the processes that did not occur in axenic flies were two hallmarks of aging conserved across animal species: the down-regulation of stress-response genes and the up-regulation of genes of the innate immune system. These processes therefore represent an adaptive response of the organism to microbes during aging.

Besides regulating gene expression, commensals also modify the fly metabolome during aging. Specifically, Yamauchi et al. discovered that allantoin, an end product of purine degradation pathway in *Drosophila*, levels increase with aging in a microbiota-dependent manner and contribute to the shortening of the lifespan (Yamauchi et al., 2020). *Acetobacter persici* was identified as a species responsible for increasing allantoin levels by activating the immune deficiency (IMD) pathway in Malpighian tubules. It remains to be discovered how increased allantoin shortens lifespan and how the IMD pathway regulates allantoin levels.

In addition to affecting the fly metabolome, commensals themselves produce metabolites that affect host aging. For example, indoles, aromatic heterocyclic organic intermediates in the biosynthesis of tryptophan, released by *E. coli* were shown to extend the lifespan of *Drosophila* by activating aryl hydrocarbon receptors (Sonowal et al., 2017). Although *E. coli* is not a typical fly gut commensal, a similar mechanism may operate in some of the *Drosophila* microbiota members that produce indoles, like *Lactobacilli*. Another bacterial metabolite, colonic acid, exhibited a similar longevity-promoting effect likely via the host’s mitochondrial dynamics regulation and unfolded protein response (UPRmt) (Han B. et al., 2017). Furthermore, a genetic approach in bacteria, a metagenome-wide association, identified bacterial methionine metabolism genes associated with the variation in *Drosophila* lifespan, suggesting a potential role of methionine in fruit fly longevity (Matthews et al., 2020).

**FIGURE 1:**
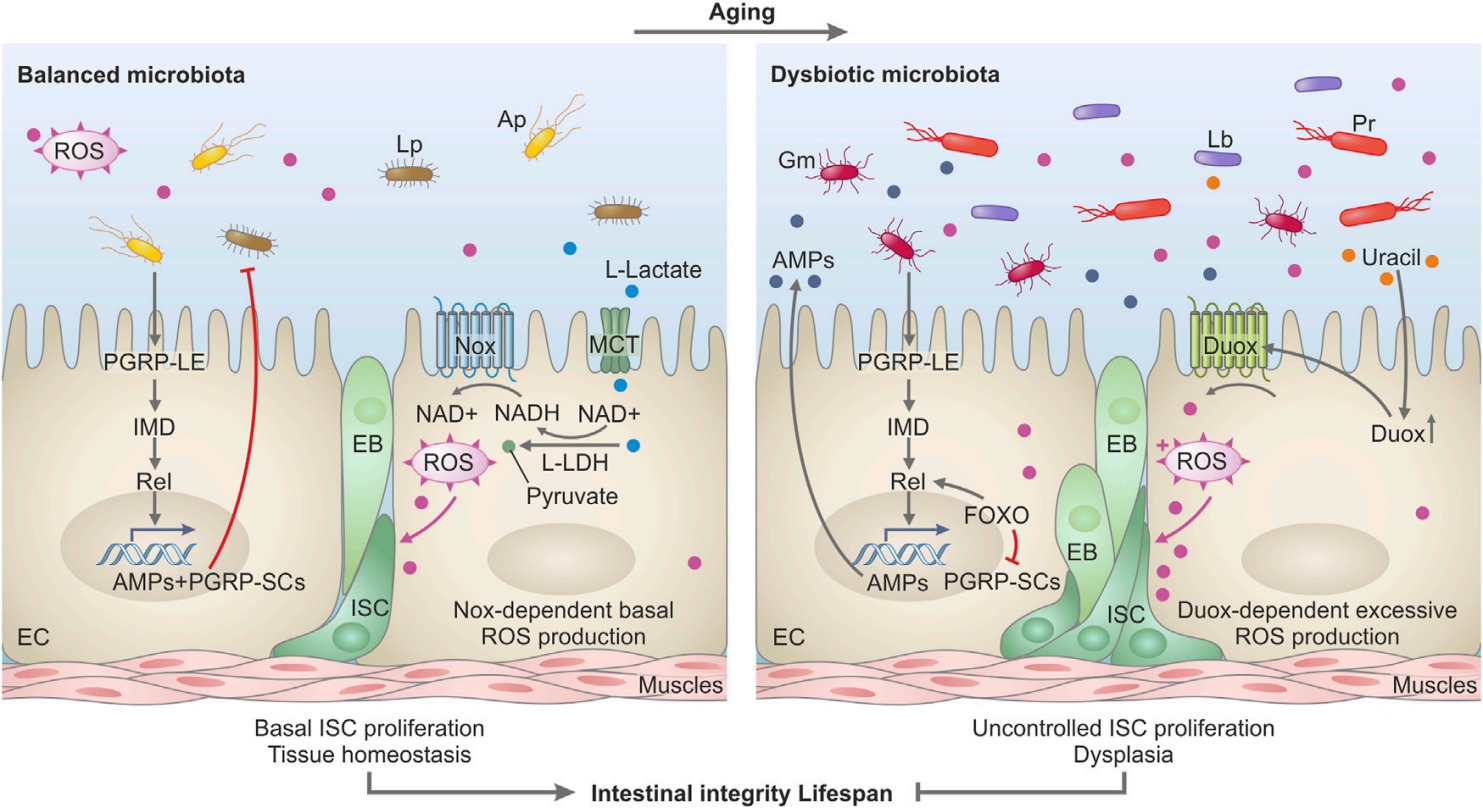
Model depicting the impact of microbiota on intestinal homeostasis during aging in *Drosophila*. In a healthy, young gut (left panel), the microbiota helps to sustain intestinal homeostasis, intestinal integrity, and optimal lifespan. Commensal bacteria like *L. plantarum* release L-lactate in the intestine. L-lactate enters the intestinal cells via monocarboxylate transporters (MCT) and is oxidized by the host lactate dehydrogenase (LDH), thus generating NADH. NADH is used by the NADPH-oxidase Nox to generate Reactive Oxygen Species (ROS) which stimulate basal intestinal stem cell (ISC) proliferation and differentiation in EnteroBlasts (EB) and Enterocytes (EC) to ensure tissue homeostasis. *L. plantarum* (Lp) and other commensals such as *A. pomorum* (Ap) stimulate basal IMD pathway activity by triggering the receptor PGRP-LE and the Relish transcription factor which consequently induces expression of PGRP-SCs and antimicrobial peptides (AMPs)—molecules that control the proliferation of the commensal bacteria. In the aging gut (right panel), the activated FOXO transcription factor boosts Relish activity and AMPs genes expression but suppresses PGRP-SCs expression. Such an environment with an excessive amount of AMPs and reduced production of PGRP-SCs favors the emergence and selection of pathobionts in the commensal community. Such pathobionts like *G. morbifer* (Gm), *L. brevis* (Lb), or *P. rettgeri* (Pr) are resistant to AMPs and release uracil which promotes excessive ROS production via activation of DUOX. DUOX-dependent ROS in turn stimulates uncontrolled ISC proliferation and defective differentiation resulting in gut dysplasia, loss of tissue integrity, and reduced lifespan.

Since the composition of the *Drosophila* microbiota changes with age and abundance increases, several studies investigated the potential causes, and consequences of such perturbations. For example, Guo et al. proposed a mechanism explaining the causes of age-related commensal dysbiosis and its impact on fly longevity (Guo et al., 2014). The authors discovered that there is a chronic activation of the FOXO transcription factor in the aging intestines. This leads to FOXO-mediated suppression of PGRP-SC2, a negative regulator of the IMD pathway, and thus to a deregulation of the activity of the IMD pathway and the induction of commensal dysbiosis in the form of increased microbial load and expansion of pathobionts (Guo et al., 2014). Consequently, such a dysbiotic microbial community caused intestinal stem cell (ISC) overproliferation, dysplasia, and reduced lifespan (Figure 1). However, how PGRP-SC2 controls microbiota, e.g., either via regulation of IMD pathway activity or by acting as an effector molecule remains to be investigated. In a subsequent study, Li et al. identified an additional mechanism behind commensal dysbiosis during aging (Li et al., 2016). Specifically, the authors found that the JAK-STAT pathway activity in the *Drosophila* gut increases with aging, likely due to the increased production of cytokines. This leads to metaplasia in the stomach-like copper cell region of the intestine. Due to its acidic pH, this region controls the distribution and composition of the microbiota. Therefore, age-related metaplasia due to increased JAK-STAT activation disrupts the copper cell region, leading to commensal dysbiosis, epithelial dysplasia, and reduced lifespan (Li et al., 2016). Thus, the decline in intestinal compartmentalization caused by age-related chronic inflammation is a crucial mechanism behind commensal dysbiosis and intestinal dysplasia.

### Link Between Host Genetics, Microbiota Dysbiosis, and Lifespan

Host genetics is one of the determinants of microbiota composition and abundance (Chaston et al., 2016). Several mutations were identified that predispose fruit flies to a shorter lifespan by affecting the intestinal microbial communities. Those mutations predominantly disrupt the host pathways implicated in the control of intestinal commensals. Fruit flies rely on two inducible defense mechanisms to control pathogens and gut microbiota: antimicrobial peptides (AMPs) and reactive oxygen species (ROS) (Ha et al., 2005; Ryu et al., 2006; Buchon et al., 2009b; Marra et al., 2021a). The dual oxidase Duox produces high levels of microbicidal ROS in response to uracil released by pathogens and pathobionts (Ha et al., 2005; Lee et al., 2013). Flies lacking Duox activity cannot control mutualistic and pathogenic bacteria and have a short lifespan (Ha et al., 2005; Ha et al., 2009). Importantly, ROS are not only microbicidal but also can damage intestinal cells, thereby inducing the compensatory proliferation of ISCs to repair the damage (Buchon et al., 2009a; Hochmuth et al., 2011). However, excessive accumulation of stem cells results in dysplasia and intestinal dysfunction.

The activation of the IMD pathway by intestinal bacteria results in the translocation of the NF-kB-like transcription factor Relish to the nucleus, which promotes the expression of antimicrobial peptides (AMPs). The IMD pathway is initiated when bacterial peptidoglycan (PGN) is sensed by the transmembrane receptor PGRP-LC in the ectodermal parts of the gut or by the intracellular receptor PGRP- LE in the midgut (Bosco-Drayon et al., 2012; Neyen et al., 2012). IMD-induced AMPs act primarily against ROS-resistant microbes and complement Duox-mediated ROS defense (Ryu et al., 2006). Flies with disrupted IMD signaling, like Relish mutants or mutants lacking AMPs exhibit an excessive load of commensals and are short-lived (Buchon et al., 2009a; Broderick et al., 2014; Iatsenko et al., 2018; Marra et al., 2021a). Several additional mutants with affected ROS- or IMD-mediated responses were shown to have reduced longevity due to commensal dysbiosis. For example, flies with reduced expression of the transcription factor Caudal have elevated IMD pathway activity and increased expression of AMPs in the gut (Ryu et al., 2008). Such an inflamed intestinal environment favors the growth of the pathobiont *Gluconobacter* EW707, which drives host mortality. A subsequent study showed that *Gluconobacter*, in contrast to beneficial microbiota members, releases uracil, which triggers chronic DUOX activation and ROS production, causing intestinal damage, uncontrolled ISC proliferation, dysplasia, and mortality (Figure 1) (Lee et al., 2013).

Loss of function of another transcription factor, Nubbin, similarly leads to a constitutively active immune response in the gut and shift in microbiota abundance and composition. Consequently, flies lacking Nubbin are short-lived due to commensal dysbiosis and overproliferation of *Acetobacter spp*. and *Leuconostoc spp* (Dantoft et al., 2016).

*Gluconobacter spp* was implicated in the intestinal pathology in another mutant with deregulated IMD activity. Chen et al. reported that flies lacking histone demethylase KDM5 exhibit gut dysbiosis due to excessive IMD pathway activity and a reduced lifespan (Chen et al., 2019). Specifically, *kdm* mutants exhibit reduced abundance of *Lactobacillus spp* but increased abundance of *Gluconobacter spp* and *Providencia spp* (Chen et al., 2019). Interestingly, another genetic deficiency, namely that of transglutaminase, results in a similar phenotype—elevated IMD activity, reduced lifespan, and commensal dysbiosis with the expansion of *Providencia spp*. and *Acetobacter spp* (Sekihara et al., 2016). Such consistency suggests that excessive IMD pathway activity creates conditions optimal for pathobionts like *Gluconobacter* and *Providencia*. Notably, both *Gluconobacter* and *Providencia* are resistant to host AMPs and ROS, while both release uracil and induce ROS via DUOX, thus not only thriving in the inflamed intestine but also further exacerbating the inflammation, intestinal damage, and dysplasia.

Flies deficient for several negative regulators of the IMD pathway, PGRP-LB, PGRP-SCs, and Pirk, are short-lived similar to other mutants with overactive IMD pathway (Kleino et al., 2008; Lhocine et al., 2008; Paredes et al., 2011). The fact that their lifespan can be extended under germ-free conditions suggests that chronic immune activation to endogenous microbes likely causes intestinal dysfunction and early death. However, whether such flies lacking negative IMD pathway regulators experience any changes in commensal composition and abundance reported for other mutants with excessive IMD activity remains to be investigated. The same question applies to the *big bang* mutant, which, due to disrupted septate junctions in the gut, has a reduced lifespan caused by constitutive immune activation that is driven by intestinal commensals (Bonnay et al., 2013).

Beyond pathobionts, bacteria that are typically considered beneficial for flies, like *Lactobacillus*, can also cause intestinal pathologies during aging when their abundance is not controlled (Fast et al., 2018; Iatsenko et al., 2018). During aging, such uncontrolled growth was observed in flies lacking PGRP-SD—a secreted receptor upstream of the IMD pathway (Iatsenko et al., 2016, 2018). Overgrowth of *L. plantarum* in the intestines of *PGRP-SD* mutant flies was accompanied by the accumulation of lactic acid, which triggered the generation of elevated levels of ROS via the intestinal NADPH oxidase Nox. Nox-generated ROS consequently caused intestinal damage, compensatory overproliferation of ISCs, dysplasia, and shortened lifespan (Iatsenko et al., 2018). The fact that *L. plantarum* induces Nox-dependent ISC proliferation in the mammalian intestine (Jones et al., 2013) suggests a conserved mechanism of Nox activation by lactate which couples bacterial growth to ISC proliferation.

Additionally, commensals might affect the host aging by modulating the key longevity-controling pathways. Indeed, mTOR and insulin-like growth factor signaling are evolutionary conserved pathways known to be regulated by fruit fly commensals (Fan et al., 2018; Bana and Cabreiro, 2019). For example, pyrroloquinoline quinone–dependent alcohol dehydrogenase (PQQ-ADH) activity of a commensal, *A. pomorum*, modulates IIS in *Drosophila* and is necessary to promote larval growth on a low-yeast diet (Shin et al., 2011). Similarly, *L. plantarum*, promotes growth of larvae on low-nutrient diet via up-regulation of the TOR pathway. Flies overexpressing the inhibitor of TOR complex 1 are resistant to the effects of *L. plantarum* on growth (Storelli et al., 2011). Given that suppression of TOR and IIS is known to promote longevity (Bana and Cabreiro, 2019), it will be important to determine whether *A. pomorum* and *L. plantarum* might negatively affect aging by activating IIS and TOR pathways respectively. Interestingly, chemical inhibition of TOR by rapamycin treatment altered microbiome composition and extended the lifespan but both in CR and axenic flies, suggesting that rapamycin’s lifespan-promoting effect is independent of microbiota (Schinaman et al., 2019).

Although Toll pathway doesn’t play a major role in intestinal immunity (Broderick et al., 2014), its role in the microbiota control is emerging. For example, Toll-deficient flies lacking PGRP-SA or DIF have reduced bacterial load and shortened lifespan. Mechanistically, PGRP-SA regulates microbiota via metabolic rather than immune function. Specifically, Toll pathway activation in enterocytes following the recognition of bacteria by PGRP-SA receptor keeps increased transcription of translational regulation factor 4E-BP. Toll activated 4E-BP enables fat catabolism, thus sustaining the microbiota. In the absence of Toll pathway activity, TOR-mediated suppression of 4E-BP results in intestinal lipid accumulation and inaccessibility which correlates with loss of intestinal bacterial density (Bahuguna et al., 2022). However, whether the reduced lifespan of *PGRP-SA* and *dif* mutants is due to lipid accumulation or changes in microbiota communities is an open question.

Similar to overactivation of IMD pathway, chronic stimulation of Toll pathway by PGN released by Gram-positive microbiota results in the short lifespan of flies. Namely, flies deficient for Kruppel-like factor 15 (Klf15) lack nephrocytes and not able to filter the hemolymph, thus accumulating microbiota-derived PGN in the hemolymph. This creates a chronic state of systemic Toll pathway activation by the PGN and shortened lifespan (Troha et al., 2019). In summary, both constitutive activation and deficiency in the major immune pathways disrupts host-commensal homeostasis leading to premature death of the host (Table 1).

**TABLE 1:** Effect of microbiota on *Drosophila* lifespan.

Treatment	Effect on Microbiota	Effect on Lifespan	References
Germ-free	Depleted/eliminated	Decreased	Brummel et al. (2004)
Germ-free	Depleted/eliminated	No change	Ren et al. (2007)
Germ-free	Depleted/eliminated	Extended	Guo et al. (2014); Petkau et al. (2014); Clark et al. (2015); Galenza et al., (2016); Li et al. (2016); Lee et al., (2019); Shukla et al. (2021)
Low-nutrient diet + monoassociation with *L. plantarum*		Extended	Téfit and Leulier, (2017)
Low-nutrient diet + monoassociation with *I. orientalis*		Extended	Yamada et al. (2015); Keebaugh et al., (2018)
Rich-nutrient diet + monoassociation with *I. orientalis*		Decreased	Keebaugh et al. (2019)
Exposure of larvae to oxidants	Elimination of *Acetobacter* species	Extended	Obata et al. (2018)
Germ-free + monoassociation with *E. coli* K12 or K12ΔtnaA		Extended in K12 vs. K12ΔtnaA, indoles are required	Sonowal et al. (2017)
*Duox* RNAi (reduces ROS)	Increase of microbiota load	Decreased	(Ha et al., 2005, 2009)
*Relish* deficiency (IMD pathway/AMP response blocked)	Increase of microbiota load	Decreased	Buchon et al. (2009a); Iatsenko et al., (2018)
*Caudal* deficiency (enhanced AMP response)	Expansion of *Gluconobacter* sp. strain EW707	Decreased	Ryu et al. (2008)
*Nubbin* deficiency (enhanced AMP response)	Expansion of *Acetobacter* spp. and *Leuconostoc* spp.	Decreased	Dantoft et al. (2016)
*Kdm5* deficiency (enhanced AMP response)	Expansion of *Gluconobacter* spp. and *Providencia* spp.	Decreased	Chen et al. (2019)
*PGRP-SD* deficiency (reduced AMP response)	Expansion of *L. plantarum*	Decreased	Iatsenko et al. (2018)
*PGRP-SA* and *Dif* deficiency (Toll pathway blocked)	Decrease of microbiota load	Decreased	Bahuguna et al. (2022)

## Discussion

In recent years substantial progress has been made in our understanding of the molecular mechanisms of microbiota influence on the fruit fly lifespan. While we have advanced our knowledge on host-microbiota interactions during aging, many essential questions remain to be addressed. For example, most studies focused on changes in bacterial communities during aging and the impact of particular bacteria on *Drosophila* lifespan. Yet, the role of other microbiome residents, like fungi and viruses, in the aging process has not been investigated and represents an exciting avenue for future studies. Similarly, fruit fly endosymbionts, like *Wolbachia* and *Spiroplasma*, have dramatic effect on the lifespan (Maistrenko et al., 2016; Marra et al., 2021b). The results, however, are inconsistent and lack mechanistic explanation. While *Wolbachia* is known to interfere with the longevity-modulating pathways (Ikeya et al., 2009; Maistrenko et al., 2016), it also modifies the intestinal microbiome via yet to be discovered mechanism (Simhadri et al., 2017). Whether such microbiome remodeling contributes to the effect of *Wolbachia* on *Drosophila* lifespan requires further investigation.

Another critical aspect that needs additional insights is the contribution of microbiota to sex differences in longevity. While it is known that *Drosophila* males live shorter than females and that there are differences between sexes in age-related intestinal pathologies and gene expression (Austad and Fischer, 2016; Hudry et al., 2016; Regan et al., 2016; Belmonte et al., 2020), the microbiome’s contribution to these differences is largely unknown. Although males and females differ in microbiota composition (Han G. et al., 2017; Leech et al., 2021), it has to be studied whether sexual dimorphism in microbiome composition and abundance or age-related microbial dysbiosis contributes to the observed pathologies and lifespan differences. An alternative possibility that needs to be tested is that the host might respond to the same commensal microbes in a sex-dimorphic manner. Thus, the same commensals might trigger different phenotypes in males and females. To address this complex question, future studies should include sex as a variable to investigate the impact of the microbiome on the aging process.

There is plenty of evidence that the composition of the diet affects various physiological processes in the host, including aging (Tatar et al., 2014; Stefana et al., 2017). Considering that the nutritional environment also dictates microbiome composition, the effect of diet on the host could also be indirectly mediated via an altered microbiome (Harris et al., 2019; Ghosh et al., 2020; Sanchez-Morate et al., 2020). Numerous studies investigated how macronutrients like proteins, lipids, carbohydrates, and their ratios affect the fruit fly physiology and commensal populations (Woodcock et al., 2015; Galenza et al., 2016; Jang and Lee, 2018; Evangelakou et al., 2019; Keebaugh et al., 2019). However, the role of micronutrients and particularly dietary transition metals remains understudied, despite their crucial importance in insect physiology (Dow, 2017; Missirlis, 2021) and interactions with pathogenic and symbiotic microbes (Iatsenko et al., 2020; Hrdina and Iatsenko, 2022). This raises the necessity to investigate how trace metals affect fly aging directly and indirectly via altering microbiome composition and function. Indeed, studies in mammals support the notion that trace metals modulate the microbiome and thus host-microbe interactions and animal health (Lopez and Skaar, 2018). Whether this is also applicable to the *Drosophila* microbiome and aging remains to be investigated.
